# Clinical Significance and Epidemiology of NO-1, an Unusual Bacterium Associated with Dog and Cat Bites

**DOI:** 10.3201/eid0802.010139

**Published:** 2002-02

**Authors:** Robyn M. Kaiser, Robert L. Garman, Michael G. Bruce, Robbin S. Weyant, David A. Ashford

**Affiliations:** National Center for Infectious Disease, Centers for Disease Control and Prevention, Atlanta, GA USA

**Keywords:** zoonotic, bite wound, CDC Nonoxidizer Group 1 (NO-1)

## Abstract

From 1974 to 1998, 22 isolates of an unusual bacterium, designated as CDC group nonoxidizer 1 (NO-1), were sent to the Centers for Disease Control and Prevention for identification. The organism's phenotypic characteristics were similar to asaccharolytic strains of *Acinetobacter*, but differed in their cellular morphology and cellular fatty acid profile. We report here on NO-1's clinical and epidemiologic significance. In all cases, isolates were recovered from an animal bite wound; 17 (77%) were isolated from a dog bite wound, 4 (18%) from a cat bite wound, and one (5%) from an unspecified animal bite. Clinical data were retrieved and reviewed for 12 (55%) of the 22 bite victims. None of the patients had pre-existing conditions associated with immunosuppression. Seven (58%) patients were hospitalized for a median stay of 5 days (range, 2 to 11 days). The median time between bite to the worsening of symptoms was 17.5 hours (range, 3 to 78 hours). All patients recovered following antibiotic treatment.

An estimated 4.4 million animal bites occur each year in the United States (*1**,**2*). The annual incidence of dog and cat bites has been reported as 300 bites per 100,000 population (*3*). The most common organisms isolated from infected dog and cat bite wounds are *Staphylococcus* spp., *Streptococcus* spp., *Corynebacterium* spp., anaerobes, and *Pasteurella multocida* (*4**,**5*).

In December 1974, the Special Bacteriology Reference Laboratory (SBRL) in the Meningitis and Special Pathogens Branch, Centers for Disease Control and Prevention (CDC), received a bacterial isolate recovered from a cutaneous bite wound of an 8-year-old boy in Texas. The isolate was a unique, fastidious, non-oxidative, gram-negative rod and was named nonoxidizer group 1 (NO-1). From 1974 to 1998, the SBRL identified 21 additional isolates received from state and local hospital laboratories across the United States and Canada. The biochemical and phenotypic characteristics of the organism have been described elsewhere (*6*). However, clinically and epidemiologically relevant data accompanying these specimens were mostly limited to source of specimen, date of collection, and occasionally sex and age of the patient. Clinical course descriptions of patients infected with NO-1 have not been previously described. To grasp the significance of this recently identified group, we conducted a review of clinical records for patients from whom NO-1 was isolated and submitted to SBRL for identification between 1974 and 1998.

## Methods

Inclusion was limited to those patients from whom isolates sent to SBRL were identified as NO-1 between 1974 and 1998. In collaboration with state and territorial public health epidemiologists and hospital infection control practitioners, we gathered relevant clinical records and exposure data to determine the clinical significance of and risk factors for NO-1 infection. State epidemiologists, hospital infection control practitioners, or both were notified of cases that occurred in their state or hospital and were requested to submit clinical records for each case, including chart notes, discharge summaries, and clinical laboratory results. Using a standardized form, we abstracted clinical data for each patient. Information recorded included demographic characteristics, signs and symptoms of illness, treatment, laboratory results, and outcome. We collected information on symptoms including erythema, swelling, drainage, cellulitis, loss of motion, and any other symptoms that treating physicians noted.

This protocol was determined to be exempt from human subjects review requirements because the gathering of clinical information to accompany strains was a standard part of reference diagnostic submission, and, therefore, considered surveillance activity for a newly emerging pathogen. No specific research question was investigated.

Data were entered and analyzed with Epi Info version 6.04 (CDC, Atlanta GA). Continuous variables were evaluated by using the Kruskal-Wallis test, and categorical variables were compared using the chi-square test or Fisher’s exact methods.

## Results

Of the 22 patients from whom NO-1 isolates were recovered, 11 (50%) were male with a median age of 22 years (range, 20 months to 78 years). The median age of males was significantly different from that of females (9 vs. 25 years, p<0.05). In all 22 cases, the organism was isolated from an animal bite wound site: 17 (77%) from a dog bite wound, 4 (18%) from a cat bite wound, and 1 (5%) with no recorded bite source. Fourteen (64%) isolates came from bites on the extremities, 6 (27%) came from face or neck wounds, and for 2 (9%) no anatomic site was specified.

We were unable to retrieve 10 (45%) of the 22 medical records for reasons including 1) records were lost, destroyed by accident or natural occurrence, or archived off-site, or 2) a patient was seen in the emergency room and notations were not in the patient record. The 12 patients whose records were available for review are summarized in Table 1. Of these patients, 4 (33%) were male with a median age of 36 years (range 7 to 78 years). Eight (67%) had dog bite wounds, and the remaining 4 (33%) had cat bites. Eleven (92%) received bite wounds on the extremities, including hands, arms, or legs; one bite occurred on the face.The median time interval from bite to onset or worsening of symptoms was 17.5 hours (range 3 to 78 hours). Ten patients were afebrile; in two cases, a temperature >38.9°C was recorded. At treatment, 67% of patients reported increased pain associated with erythema and swelling around the wound site. Purulent drainage was noted in 50% of cases, and a diagnosis of cellulitis was made for 67% of patients. There was no evidence of sepsis, although blood cultures were attempted in only four cases. For those patients for whom records were retrieved, decreased range of motion was documented in 4 (36%) of the 11 with extremity wounds. Other reported symptoms included ecchymosis (8%), increased local temperature (8%), tingling sensations (8%), chills (8%), adenitis/lymphadenopathy (25%), and discoloration (25%) including “streaking.” Total leukocyte counts ranged from 8,100 to 12,700 cells/mm^3^. Hematocrit (range 36% to 43%) and platelet counts (range 140 to 331 K/mm^3^) were normal. Laboratory reports on cultures from wound specimens are summarized in Table 2. Isolates later identified as NO-1 were originally described as fastidious gram-negative rods, polymorphic gram-negative rods, or gram-negative bacilli. Concentrations of NO-1 in these specimens ranged from light to moderate. Most of the specimens contained mixtures of two or more bacterial species, although specimens from Cases 6 and 11 yielded apparently pure cultures of NO-1. Other bacterial organisms isolated from these wounds included *Weeksella zoohelcum*, *Eikenellla corrodens*, *Pasteurella multocida*, *Staphylococcus aureus*, and *Corynebacterium* spp., coagulase-negative *Staphylococci*, and unidentified gram-positive cocci and enteric gram-negative rods. None of the NO-1 isolates were identified by the original hospital laboratory.

**Table 1:** Characteristics of patients for whom records were available and from whom NO-1 isolates were obtained

Case no.	State	Gender/ age (yrs)	Animal	Wound site	No. days ill	Sutured	Major clinical features	Antibiotic treatment/outcome
1	MA	F/22	Cat	Hand	8	Unk.	Cellulitis, loss of motion, drainage, fever	Improved with cephradine + penicillin (OP)
2	IL	M/45	Dog	Hand	5	Unk.	Purulent drainage, redness and swelling extending up to forearm	Cefadroxil (OP); next day admitted to hospital and gradual improvement with ampicillin/sulbactam (IP)
3	IL	F/11	Dog	Hand	6	Yes	Cellulitis, discharge, local increase in temperature	Improved with ampicillin + ceftriaxone, local bacitracin (IP)
4	RI	F/78	Cat	Hand	3	Unk.	Cellulitis, decrease movement	Antibiotic ointment + amoxicillin/clavulanic acid (OP1); penicillin (OP2); admitted to hospital next evening and gradually improved on ampicillin/sulbactam (IP)
5	SC	F/37	Dog	Hand	7	Unk.	Cellulitis, purulent drainage	Cephradine (OP); admitted to hospital and improved on gentamicin + ampicillin (IP)
6	GA	M/7	Dog	Leg	11	Yes	Cellulitis, profuse drainage, fever, red streaking, inguinal adentitis, chills	Amoxicillin (OP); improved on penicillin + cefazolin, changed to cephalexin (IP)
7	MA	M/61	Dog	Arm	Unk.	Unk.	Superficial laceration with streaking	Penicillin^a^ (OP)
8	CA	M/35	Dog	Hand	3	Yes	Cellulitis, loss of motion	Iproved with cefazolin + cephradine (OP)
9	CA	F/24	Cat	Hand	2	Unk.	Cellulitis, purulent discharge, lymphadenitis, tingling sensations	Resolved with cefazolin + penicillin (IP);
10	TN	F/51	Dog	Face	5	Yes	Pustule of wound	Improved with cephalexin (OP)
11	NV	F/56	Cat	Arm	Unk.	Unk.	Swelling, lymphadenopathy	Dicloxacillin^a^ (OP)
12	MI	F/21	Dog	Hand	3	Yes	Cellulitis, impaired range of motion	Cefaclor (OP) + improved with cefazolin (IP)

Unk. = Unknown; OP = outpatient; OP1 = outpatient visit 1; OP2 = outpatient visit 2; IP = inpatient.

^a^Incomplete information on condition of infection following treatment.

**Table 2:** Description of all bacterial isolates cultured from infected wounds

Case no.	Bacterial organisms cultured
**1**	No documentation of culture results in record
**2**	Light Weeksella zoohelcum; light fastidious gram-negative bacilli^a^
**3**	Few fastidious gram-negative rods^a^; coagulase-negative Staphylococci on subculture only
**4**	No growth of organisms noted in record
**5**	Enterics, few gram-positive cocci; some polymorphic gram-negative rods^a^
**6**	Light growth of gram-negative bacilli^a^
**7**	No documentation of culture results in record
**8**	Three types of gram-negative rods^a^
**9**	Few unidentified gram-negative rods^a^; few mixed aerobic skin flora
**10**	Rare Eikenella corrodens; few gram-negative bacilli^a^
**11**	Moderate growth of gram-negative bacilli^a^
**12**	Numerous Pasteurella multocida; rare Staphylococcus aureus; few gram-negative rods^a^; numerous Corynebacterium species

^a^Identified as Centers for Disease Control and Prevention group nonoxidizer 1 (NO-1).

No data were available on the depth of the wounds. Standard cleansing, including irrigation and debridement of wounds followed by dressing, was documented for 8 of 12 patients. Five of the 12 patients had wound closure by suture after irrigation. No associations were seen between suture therapy and severity of symptoms or duration of recovery.

Seven (58%) persons were hospitalized for treatment, 5 (42%) were managed as outpatients (Table 1). All 12 patients were given one or more antibiotic treatments within 1 to 3 days of receiving bite wounds. Initial therapy for patients included at least a beta-lactam antibiotic (Table 1). Five patients (Cases 2, 4, 5, 6, and 12) were initially treated as outpatients and were subsequently admitted for worsening of symptoms. In-hospital treatment for these five patients included either intravenous beta-lactam or intravenous beta-lactam with beta-lactamase inhibitor. One patient also received an aminoglycoside. Eight (67%) of 12 cases, including all 7 inpatients, received intravenous antibiotic therapy. Four of the five outpatients received oral antibiotics only. However, no documentation of compliance for these patients was found. Though most patients were released before infection completely resolved, all 10 patients for whom follow-up information was available had documented improvement of the infection. Symptoms resolved within 2 to 11 days (median 5.5 days). The hospitalized patients had a median stay of 4 days (range 2 to 11 days).

A history of asthmatic bronchitis and respiratory allergies, arthritis, and a ventricular septal defect was noted for Cases 6, 4, and 9, respectively. Two patients were elderly (>60 years). No pre-existing conditions or illnesses were documented for the rest of the patients.

## Discussion

NO-1 is a recently identified bacterium associated with dog and cat bite wounds. Infections in which NO-1 bacteria were isolated appear to be local (i.e., abscess and cellulitis). Following receipt of a bite wound, NO-1 infections, without severe disease, can occur in healthy persons with no underlying illness. The most common clinical features associated with NO-1 infections included purulent drainage, increased pain with erythema and swelling, and cellulitis, which are clinically similar to features of infections caused by other animal bite-related organisms (*5*). Apparently pure cultures of NO-1 were obtained from wound specimens of Cases 6 and 11. Although Case 11 resolved with outpatient dicloxacillin treatment, Case 6 required hospitalization and multiple antibiotic treatments. This patient also had symptoms consistent with septicemia (fever, chills). Taken together, these facts suggest that NO-1 infections can progress from localized to systemic forms. At this time there is no available information on potential virulence factors for NO-1.

As described by Hollis, et al., the phenotypic characteristics of NO-1 are similar to those observed with asaccharolytic *Acinetobacter* species (*6*). NO-1 bacteria fail to acidify carbohydrates and are oxidase, indole, and urease negative. Cellular fatty acids and ubiquinone analysis are useful in differentiating NO-1 from *Acinetobacter* species (*6*)*.* Studies are under way at the SBRL to determine the taxonomic classification of NO-1.

The patients from whom NO-1 were isolated were successfully treated with a variety of antibiotics. In general, intravenous antibiotics my have an advantage over oral antibiotics in preventing bacterial infections associated with bite wounds, including those caused by NO-1, because of more rapid delivery of drug to affected tissues (*7*). Previously reported antimicrobial susceptibility testing of 17 of the 22 strains by a standard broth microdilution method showed all stains to be susceptible to aminoglycosides, beta-lactam antibiotics, tetracyclines, quinolones, and sulfonamides. Fifty percent were resistant to trimethoprim. Some of the isolates were noted to grow poorly in the broth test for the antimicrobial susceptibility testing, but all control wells had sufficient growth for interpretable results (*6*).

Most dog and cat bite wounds in young children occur on the face, head, and neck; by contrast, the extremities tend to be injured in young adults and adults (*2**,**8**,**9*). In our study, of the 6 patients who received bite wounds to the face, head, or neck, 4 (67%) were ≤8 years, 1 (17%) was a 51-year-old woman, and 1 (17%) was of unknown age. Of the 14 victims who received bite wounds on an extremity, 1 (7%) was <10 years. Across all ages, 14 (64%) of all 22 isolates came from bite wounds on the extremities, consistent with the distribution of animal bite wounds reported in other studies (*5**,**10**,**11*).

In addition to other pathogens that are associated with bite wounds, such as *Staphylococcus* spp., *Streptococcus* spp., *Corynebacterium* spp., anaerobes, and Pasteurella multocida, NO-1 should be considered in the differential diagnosis of infected bite wounds from cats and dogs. Group NO-1 organisms have been shown to be similar to two isolates from oral and nasal fluids from dogs called “unidentifiable species no. 4” by Bailie et al. (*12*). Likewise, the association of human NO-1 infections with animal bite wounds received from cats and dogs suggests that these animals are a reservoir for NO-1 bacteria.

At present no surveillance system exists for reporting dog or cat bite wound-associated infections and, therefore, the incidence of NO-1 isolated from bite wound infections is not known; however, these 22 reported cases likely represent only a fraction of the true number of potential NO-1 infections. Several of the case records noted the gram-negative rods as possible exogenous contaminants; thus, many more NO-1 infections may be unrecognized and undocumented. The etiologic role and the pathogenicity of NO-1 are unclear. Enhanced awareness of this organism by physicians will improve our understanding of this new zoonotic infection and clarify its clinical significance. With over 4 million animal bites occurring in the United States each year, bacteria associated with dog and cat bite wounds, including NO-1, are an important public health problem.
